# Vaccination strategies, the power of the unmatched double hits

**DOI:** 10.2144/fsoa-2023-0090

**Published:** 2023-07-06

**Authors:** Talal S Al-Qaisi, Berjas Abumsimir

**Affiliations:** 1 Department of Medical Laboratory Sciences, Pharmacological & Diagnostic Research Centre (PDRC), Faculty of Allied Medical Sciences, Al-Ahliyya Amman University (AAU), Amman, 19328, Jordan

**Keywords:** heterologous vaccination, homologous vaccination, immune dominance, prime-boost vaccine

This commentary is set out to highlight the comparison between heterologous versus homologous vaccination strategies and to provide a brief scientific discussion of the history and background of the heterologous vaccination practice. In addition, we focus on the advantages of heterologous vaccination from an immunological perspective as a contribution to the global efforts for optimization and standardization of vaccines to be prepared for future vaccine development.

Vaccines employ pathogenic antigen-derived products to assist the immune system response promptly once the antigen is met throughout the body. To respond properly, the immune system usually has to meet the foreign antigen more than once to generate memory. When it comes to vaccination techniques, the first injection is known as a priming vaccine, while the second and third shots are known as booster vaccines. For example, to develop appropriate anti-HBs levels, homologous vaccination using recombinant hepatitis B (HB) protein can be administered in two or three doses using the same vaccine for priming and boosting [1].

Booster vaccines could be homologous or heterologous. When an individual is exposed to a certain antigen carried by the same vector more than once, this is called homologous boost, the immune response is generated against all epitopes (including both the vector and the antigen of interest). In heterologous boost, the individual is exposed to the same antigen more than once, but each time the antigen of interest is carried by a different vector, this allows the immune system to respond preferentially to the epitopes shared with the original vaccine (which are the epitopes of the antigen of interest), and makes a smaller than normal response to the epitopes on the vector.

With the progression of vaccine development, innovative vaccination platforms such as DNA vaccines, mRNA vaccines, viral vector-based vaccinations and conjugate vaccines are being used in clinical trials and clinics. Interestingly, multiple studies have found that heterologous vaccination tactics that utilize DNA vaccines for priming and protein vaccines for boosting, or *vice versa*, can elicit higher humoral and cellular immune responses than homologous vaccination strategies.

Although most immunization programs employ homologous vaccination strategies, new research suggests that heterologous vaccines can be as effective as or more effective than homologous vaccinations. By presenting the antigen of interest in the setting of another vaccination platform, heterologous booster vaccines are expected to increase the immunological response induced by the primer vaccine [2]. Combining two different vaccination platforms against the same target has frequently been demonstrated to be more successful than each one alone in triggering an effective immune response and creating a long-term memory response, allowing for a stronger and longer-lasting immune response [3]. Cancer immunotherapy [4], HIV prevention [5], COVID-19 [6] and influenza vaccine [7] have all employed the heterologous prime and boost technique.

This topic garnered a lot of attention during the COVID-19 campaign, mainly because of the increasing demand for vaccinations and the scarcity of supply. As a result, researchers investigated the idea of employing alternative vaccinations for priming and boosting to compensate for the vaccine supply constraint. However, study in this area is not new, as academics investigated this concept in the early 1980s [8].

In the field of HIV prevention, heterologous prime and boost tactics have been utilized to create vaccines that can protect against different strains of HIV at the same time. These vaccinations often comprised antigens from many strains of HIV, which were subsequently enhanced with antigens from other strains. This method has been found to be more successful than single-strain vaccinations in protecting against many strains of HIV.

Emini *et al.* employed synthetic peptides for priming the immune response against poliovirus followed by a booster of inactivated polio virus in small animal trials in 1983 and reported production of neutralizing antibodies against the intact virus [9]. Hu *et al.* discovered in 1991 that priming rats with a live recombinant HIV virus and boosting with a subunit recombinant protein was more efficient than either immunogen alone [10]. In 1992, the same group reported efficient protection of non-human primate models by using heterologous prime-boost immunization in the context of developing an AIDS vaccine [11]. Girard *et al.* observed a substantial rise in antibody titers in a chimpanzee primed with a recombinant vaccinia virus and boosted numerous times with a combination of recombinant HIV-1 proteins or synthetic peptides [12]. Daniel Zagury conducted an experiment of heterologous prime-boost vaccination, inoculating himself with a recombinant vaccinia virus encoding the HIV-1 Env gene and then boosting with a recombinant Env protein, this study showed for the first time that immunity can be generated in humans against the HIV virus [13].

In influenza vaccination programs, heterologous prime and boost techniques have also been deployed. A single dose of a ‘prime’ influenza vaccine is provided in this application, followed by a ‘boost’ injection containing additional antigens from other strains of influenza virus several weeks later. This method has been found to be more effective than single-strain vaccinations in protecting against various strains of influenza [14].

Some of these prime-boost combinations have advanced to clinical trials, where they have shown encouraging benefits. In investigations on Ebola virus vaccines, for example, heterologous immunization demonstrated a robust and prolonged augmentation of specific immunity without causing any vaccine related significant side effects [15].

Importantly, the heterologous prime-boost regimen is more immunogenic than the homologous prime-boost regimen in many vaccines, while the specific mechanism is unknown. It is hypothesized that DNA or mRNA-based vaccines will result in the production of memory B cells specific for the antigen’s conformational domains; this is supported by Vaine *et al.*, who demonstrated the presence of conformational sensitive antibodies after priming with a DNA-based vaccine for *in vivo* delivery of HIV-1 gp120 antigen [16].

Schmidt *et al.* found that a heterologous vaccine regimen induced spike-protein-specific IgG, neutralizing antibodies and spike-protein-specific CD4^+^ T cells, all of which were significantly higher than after a homologous vector vaccine boost and comparable to homologous mRNA vaccine regimens. Furthermore, spike-protein-specific CD8^+^ T cell numbers were considerably greater following heterologous immunization than after both homologous regimens [17]. Larger countrywide cohort trials found equal vaccination effectiveness against SARS-CoV-2 infection following a heterologous ChAdOx1/mRNA-based COVID-19 vaccine prime-boost compared with a homologous mRNA-based COVID-19 prime-boost [18].

Furthermore, there is some intriguing evidence that a heterologous prime-boost regimen may aid immunocompromised persons in mounting a greater immune response than a homologous regimen [19]. Based on these factors, the CDC’s Advisory Committee on Immunization Practices (ACIP) released guidelines in October 2021 in favor of the ‘mix-and-match’ heterologous increase COVID-19 vaccination approach [20].

A heterologous COVID-19 prime-boost vaccination regimen against SARS-CoV-2 infections is both safe and efficacious. Heterologous prime-boost regimens may produce similar or greater antibody (spike protein) titers than homologous prime-boost regimens. The reactogenicity profile of heterologous prime-boost was identical to that of homologous prime-boost. In immunocompromised patients, heterologous prime-boost immunization may help produce a higher immune response.

In a ‘heterologous’ prime-boost configuration, researchers have demonstrated that prime-boost vaccinations may be administered using mismatched vaccine delivery mechanisms while employing the same antigen. The most intriguing and unexpected discovery is that, in many circumstances, heterologous prime-boost outperforms ‘homologous’ prime-boost. During our fight against COVID-19, the quick acceptance and deployment of innovative vaccination techniques has undoubtedly broadened the scope of heterologous prime-boost immunization.

In the case of COVID-19 vaccines, the concept of heterologous vaccination came to the surface, by using a variety of platforms, including nucleic acid (DNA and RNA), viral vectors, protein subunits and whole-virus platforms. For instance, the mRNA vaccines (BNT162b2 Pfizer BioNTech, mRNA-1273 Moderna) encode for the SARS-CoV-2 spike protein, viral vector vaccines (ChAdOx1 AstraZeneca/Oxford, Janssen/Ad26.COV 2.S) use adenovirus-based vectors, and inactivated whole-virus vaccine (Sinopharm, Sinovac-CoronaVac) use the whole virus to deliver genetic material (encoding for SARS-CoV-2 spike protein) to host cells.

Immune dominance, in our opinion, is significant in heterologous prime and boost effectiveness because it allows the immune system to focus its response on the most often encountered antigens. This is because the antigen of interest is given to the immune system in diverse settings when a heterologous prime and boost technique is applied. This enables a more targeted immune response to the antigen of interest rather than the backbone, carrier protein or vector. This may result in a more effective vaccination mechanism than homologous techniques, which show all antigens repeatedly and dilute the immune response to the antigen of interest.

Overall, heterologous prime and boost techniques have been shown to be quite successful in eliciting significant immune responses to numerous infections or antigens. This method has been used effectively in many different fields of medicine and is still being researched for possible uses in other areas.

We urge that a systematic study is conducted to determine if the immunization order affects the effectiveness of heterologous vaccination. More basic research is also required to apply what we know about heterologous vaccination to develop a more protective immune response against less dominant antigens, which may be more protective against some infections.
